# Prostate cancer cells elevate glycolysis and G6PD in response to caffeic acid phenethyl ester-induced growth inhibition

**DOI:** 10.1186/s12885-025-13477-6

**Published:** 2025-01-16

**Authors:** Tzu-Ping Lin, Pei-Chun Chen, Ching-Yu Lin, Bi-Juan Wang, Ying-Yu Kuo, Chien-Chih Yeh, Jen-Chih Tseng, Chieh Huo, Cheng-Li Kao, Li-Jane Shih, Jen-Kun Chen, Chia-Yang Li, Tzyh-Chyuan Hour, Chih-Pin Chuu

**Affiliations:** 1 Faculty of Medicine, National Yang Ming Chiao Tung University, Hsinchu City, 30010 Taiwan; 2 Department of Urology, Taipei Veterans General Hospital, Taipei City, 11217 Taiwan; 3 Institute of Cellular and System Medicine, National Health Research Institutes, Miaoli County, 35053 Taiwan; 4 Ph.D. Program for Cancer Molecular Biology and Drug Discovery, Taipei Medical University, Taipei City, 11031 Taiwan; 5 Department of Education and Medical Research, Taoyuan Armed Forces General Hospital, Taoyuan City, 325208 Taiwan; 6 Division of Colon and Rectal Surgery, Department of Surgery, Tri-Service General Hospital, National Defense Medical Center, Taipei City, 114202 Taiwan; 7 Immunology Research Center, National Health Research Institutes, Miaoli County, 35053 Taiwan; 8 Division of Urology, Departments of Surgery, Tri-Service General Hospital, National Defense Medical Center, Taipei City, 114202 Taiwan; 9 Division of Urology, Department of Surgery, Taoyuan Armed Forces General Hospital, Taoyuan City, 325208 Taiwan; 10 Graduate Institute of Medical Science, National Defense Medical Center, Taipei City, 114202 Taiwan; 11 Institute of Biomedical Engineering and Nanomedicine, National Health Research Institutes, Miaoli County, 35053 Taiwan; 12 Graduate Institute of Medicine, College of Medicine, Kaohsiung Medical University, Kaohsiung City, 80708 Taiwan; 13 Department of Medical Research, Kaohsiung Medical University, Kaohsiung City, 80708 Taiwan; 14 Neuroscience Research Center, Kaohsiung Medical University, Kaohsiung City, 80708 Taiwan; 15 Department of Biochemistry, School of Medicine, Kaohsiung Medical University, Kaohsiung City, 80708 Taiwan; 16 Department of Medical Research, Kaohsiung Medical University Hospital, Kaohsiung City, 80708 Taiwan; 17 PhD Program for Aging, China Medical University, Taichung City, 40402 Taiwan; 18 Biotechnology Center, National Chung Hsing University, Taichung City, 40227 Taiwan; 19 Department of Life Sciences, National Central University, Taoyuan City, 32031 Taiwan

**Keywords:** Caffeic acid phenethyl ester, Prostate cancer, Glycolysis, G6PD, Pyruvate, Lactate

## Abstract

**Background:**

Caffeic acid phenethyl ester (CAPE) is the main bioactive component of poplar type propolis. We previously reported that treatment with caffeic acid phenethyl ester (CAPE) suppressed the cell proliferation, tumor growth, as well as migration and invasion of prostate cancer (PCa) cells via inhibition of signaling pathways of AKT, c-Myc, Wnt and EGFR. We also demonstrated that combined treatment of CAPE and docetaxel altered the genes involved in glycolysis and tricarboxylic acid (TCA) cycle. We therefore suspect that CAPE treatment may interfere glucose metabolism in PCa cells.

**Methods:**

Seahorse Bioenergetics platform was applied to analyzed the extra cellular acidification rate (ECAR) and oxygen consumption rate (OCR) of PCa cells under CAPE treatment. UPLC-MSMS with Multiple Reaction Monitoring (MRM), PCR, and western blot were used to analyze the effects of CAPE on metabolites, genes, and proteins involved in glycolysis, TCA cycle and pentose phosphate pathway in PCa cells. Flow cytometry and ELISA were used to determine the level of reactive oxygen species in PCa cells being treated with CAPE.

**Results:**

Seahorse Bioenergetics analysis revealed that ECAR, glycolysis, OCR, and ATP production were elevated in C4-2B cells under CAPE treatment. Protein levels of glucose-6-phosphate dehydrogenase (G6PD), phosphogluconate dehydrogenase (PGD), glutaminase (GLS), phospho-AMPK Thr172 as well as abundance of pyruvate, lactate, ribulose-5-phosphate, and sedoheptulose-7-phosphate were increased in CAPE-treated C4-2B cells. ROS level decreased 48 h after treatment with CAPE. Co-treatment of AMPK inhibitor with CAPE exhibited additive growth inhibition on PCa cells.

**Conclusions:**

Our study indicated that PCa cells attempted to overcome the CAPE-induced stress by upregulation of glycolysis and G6PD but failed to impede the growth inhibition caused by CAPE. Concurrent treatment of CAPE and inhibitors targeting glycolysis may be effective therapy for advanced PCa.

**Supplementary Information:**

The online version contains supplementary material available at 10.1186/s12885-025-13477-6.

## Introduction

 Prostate cancer (PCa) is the second most frequently diagnosed cancer of men worldwide. Androgen deprivation therapy (ADT) is the standard treatment for metastatic PCa. However, numerous PCa patients receiving ADT will ultimately establish castration-resistant prostate cancer (CRPC) [1, 2]. Treatment with docetaxel plus prednisone has been approved as a first-line treatment for CRPC patients [3]. However, a significant proportion of PCa patients receiving docetaxel-based therapy eventually develop drug resistance [4, 5]. Next generation androgen signaling inhibitors abiraterone acetate and enzalutamide have been approved in recent years to treat CRPC patients. Patients receiving treatment with enzalutamide or abiraterone showed longer overall survival, better progression-free survival, and better PSA response rate [6–8]. However, 20–40% of patients do not respond to enzalutamide or abiraterone, and a significant portion of patients who initially respond to enzalutamide or abiraterone will finally acquire drug-resistant relapsed tumors within months [9–11]. Novel therapeutic agents are therefore in need for advanced PCa treatment.

The European propolis is originated from the poplar tree [12, 13]. European propolis is rich in flavonoid aglycones, hydroxycinnamic acids and their esters [14]. Caffeic acid phenethyl ester (CAPE) is the main bioactive component of poplar type propolis. CAPE is a lipophilic derivative of caffeic acid and a phenolic antioxidant structurally related to 3, 4-dihydroxycinnamic acid [15, 16]. CAPE is a NF-κB inhibitor [16]. CAPE treatment suppresses the proliferation and survival of different types of human cancer cell lines [17]. We previously reported that CAPE treatment repressed the cellular survival, proliferation, and tumor growth of both androgen-dependent and CRPC PCa cell lines via inhibition of PI3K-Akt and c-Myc signaling [18–20]. CAPE suppressed the migration and invasion of PCa cells via inhibition of Wnt signaling and EGFR signaling [21, 22]. Additionally, we demonstrated that combination treatment of docetaxel with CAPE effectively decreased the proliferation and survival of docetaxel-resistant PCa cells via reoression of Bcl-2 and c-Myc [23]. Combination treatment of CAPE and docetaxel increased the genes involved in glycolysis and tricarboxylic acid (TCA) cycle [23]. We therefore suspect that CAPE treatment may interfere glucose metabolism in PCa cells.

In normal cells, glucose is catabolized to pyruvate. Pyruvate is further converted to acetyl-CoA and oxidized via the mitochondrial TCA cycle, resulting in the production of NADH and FADH2 [24]. Glycolysis has been shown to induce drug-resistance in cancers [25]. Up-regulation of glycolysis was reported to promote drug- or therapy- resistance in colorectal cancer, breast cancer, leukemia, gastric cancer, lung cancer, and cancer stem cells [26–32]. Some chemotherapy drugs and tyrosine kinase inhibitors (TKIs) enhance the glycolysis of cancer cells. In hyperglycemic conditions, imatinib decreased the viability of PCa cells but increased both glucose consumption and lactate production in PC3 and DU-145 cells [33]. TKI erlotinib suppresses the proliferation and EGFR signaling but increases glycolytic pathway in non-small cell lung cancer cells [32]. Elevation of glycolysis may promote the drug resistance via induction of DNA repair, epithelial-mesenchymal transition (EMT), autophagy, drug efflux as well as inhibition of apoptosis and drug influx in cancer cells [25]. Additionally, alteration in glucose metabolic pathways is essential for the disease progression of PCa [34–38]. We therefore examined the effect of CAPE treatment on glucose metabolism-related signaling pathways in CRPC cells.

## Materials and methods

### Chemicals and cell culture

Caffeic acid phenethyl ester (CAPE) was purchased from Sigma Aldrich (St. Louis, MO, U.S.A) (CAS 104594-70-9). Compound C was purchased from Selleckchem (Houston, TX, USA) (Cat. S7306). The human C4-2B PCa cell line was a gift from Dr. Hsing-Jien Kung (Taipei Medical University, Taiwan). C4-2B cells were maintained in Roswell Park Memorial Institute (RPMI)1640 medium containing 10% fetal bovine serum (FBS), penicillin (100 U/mL) and streptomycin (100 µg/mL). Cells were cultured in the incubator at 37 °C with 5% CO_2_ and passaged every 3–4 days. FBS and DHT was from Biological Industries (Beit Haemek, Israel) and Sigma Aldrich (St. Louis, MO, U.S.A), respectively.

### Cell proliferation assay

C4-2B cells were seeded at a density of 3000 cells/100 µL in 96-well plates. After one day, the cells were treated with various concentrations (0, 2.5, 5, 10, 20, 40 µM) of CAPE in additional 100 µL medium for 24, 48, 72, and 96 h. Relative cell number was analyzed by measuring the DNA content of cell lysates with Hoechst dye 33,258-based 96-well proliferation assay (Sigma, St. Louis, MO, U.S.A) as described previously [39]. All readouts were normalized to the average of the control condition in each individual experiment.

### Cell viability assay

C4-2B cells were seeded at a density of 3000 cells per well in 100 µL of culture medium in 96-well plates. After a day, the cells were treated by CAPE in different concentrations (0, 2.5, 5, 10, 20, 40 µM) in 100 µL of culture medium for 48 and 96 h. Cell viability was assessed by an MTT (3,4,5-dimethylthiazol-2-yl)−2-5-diphenyltetrazolium bromide) assay as previously described [19]. The amount of formazan was determined by measuring the absorbance at 560 nm using an Tecan GENios™ plate reader (Tecan group Ltd, Männedorf, Switzerland). All results were normalized to the average of the control condition in each individual experiment.

### Seahorse bioenergetics analysis

Seahorse Extracellular Flux Analyzer (Seahorse Bioscience) was used to monitored cellular oxygen consumption rates (OCR) and extracellular acidification rates (ECAR) in real-time by Mito Stress Test and Glycolysis Stress Test, respectively. Detail of experimental procedure was described in Supplemental Materials and Methods.

### Immunoblot analysis

Western blotting was conducted as previously described [18]. Information of antibodies detecting specific proteins was listed as following: The PGD, GLS, ALDOA, LDHB, c-Myc, AMPK were from Epitomics/Abcam (Cambridge, MA, USA); G6PD, PKM2, phospho-AMPK Thr172 were from Cell signaling (Danvers, MA, USA); β-actin was from Novus (Littleton, CO, USA); SOD2 was from GeneTex (Irvine, CA, USA); PFK1, PFK2 were from Santa Cruz (Dallas, TX, USA); phospho-PFK2 Ser466 was from Invitrogen (Waltham, MA, USA).

### Real-time quantitative PCR

RNA was extracted with RNeasy Mini Kit (Qiagen, Germantown, MD, USA). The total RNA were reverse transcript to cDNA by RevertAid H minus first strand cDNA synthesis kit (Thermo Fisher Scientific, Waltham, MA, USA). Specific gene expression level were assayed using SYBR Green/ROX real-time PCR (Thermo Fisher Scientific, Waltham, MA, USA). 18 S rRNA was used as RNA content loading control [40]. The qRT-PCR primers were synthesis by using a web-based design software (Primer3: http://frodo.wi.mit.edu/primer3/), Primer sequence as following descripted: *MYC*-F: AGCTGCTTAGACGCTGGATT, *MYC*-R: TCCTGTTGGTGAAGCTAACG; *ALDOA*-F: ATGCCCTACCAATATCCAGCA, *ALDOA*-R: GCTCCCAGTGGACTCATCTG; *GAPDH*-F: AATCCCATCACCATCTTCCAG, *GAPDH*-R: CCTTCTCCATGGTGGTGAAGAC; *PDK4*-F: GAGGATTACTGACCGCCTCTTTAG, *PDK4*-R: TTCCGGGAATTGTCCATCAC; *PGAM2*-F: AGAAGCACCCCTACTACAACTC, *PGAM2*-R: TCTGGGGAACAATCTCCTCGT; *TALDO1*-F: TCGGTCTTGCTATGTCGAGC, *TALDO1*-R: TGTACTCGTCGATGGCGTG; *IDH1*-F: AGAAGCATAATGTTGGCGTCA, *IDH1*-R: CGTATGGTGCCATTTGGTGATT; *GDH*-F: GGAGATGTCCTGGATCGCTG, *GDH*-R: GTCCATGGATTCCCCCTTGG; *GLS*-F: GACATGGAACAGCGGGACTAT, *GLS*-R: TGTCCTTGGGGAAAGGGTTT; *GSR*-F: ACGGCATGATAAGGGGATTCA, *GSR*-R: AGTTTTCGGCCAGCAGCTAT; *G6PD*-F: ACAGAGTGAGCCCTTCTTCAA, *G6PD*-R: GGAGGCTGCATCATCGTACT; *PGD*-F: GTACCCGTCACCCTCATTGG, *PGD*-R: AGAGTGCCTTCCGAATGTCC. Primer and probe sequences used for PSA quantification were described by Gelmini et al. [41].

### Measurement of intracellular oxidative stress

We used the ROS indicator 6-carboxy-2’,7’-dichlorodihydrofluorescein diacetate (carboxy-H_2_DCFDA, C400; Invitrogen) to assess the intracellular ROS generation after CAPE treatment. The intracellular ROS was determined by flow cytometry and analyzed with FlowJo software. Detail experimental procedure was described in Supplemental Materials and Methods.

### Statistical analysis

Statistical analyses were performed using one-way ANOVA. Statistically significant *p* values are abbreviated as follows: *, *p* < 0.05; **, *p* < 0.01; ***, *p* < 0.001.

## Results

### CAPE treatment suppresses the cell proliferation and survival of C4-2B human PCa cells

**Fig. 1:**
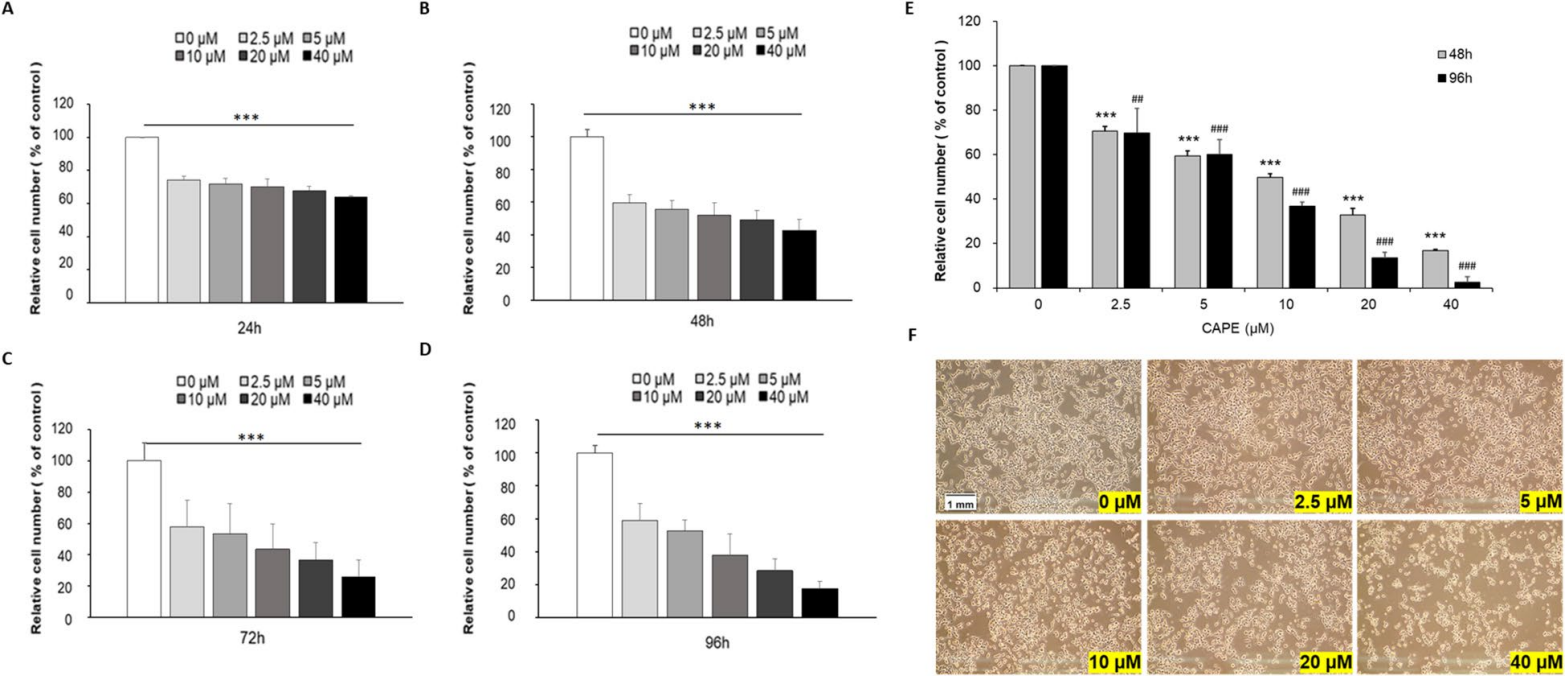
The effect of CAPE on cell proliferation of C4-2B cells. Human C4-2B PCa cells were treated with increasing concentrations of CAPE (0, 2.5, 5, 10, 20, 40 µM) for 24 h (**A**), 48 h (**B**), 72 h (**C**) or 96 h (**D**). The cell proliferation was examined by Hoechst 33,258 proliferation assay. Relative cell number of each condition was normalized to the cell number of control at different time treatment (24, 48, 72 and 96 h). Asterisks *** represented statistically significant difference *p* < 0.001 between the group and control group as determined by One-Way ANOVA. (**E**) C4-2B cells were treated with increasing concentrations of CAPE (0, 2.5, 5, 10, 20, 40 µM) for 48–96 h. Survival of C4-2B cells was determined by MTT assay. The relative cell number was normalized to the cell number of the control group at specific treatment time. *** represented statistically significant difference *p* < 0.001 between the group and control group of 48 h treatment. ## and ### represented statistically significant difference *p* < 0.01 and *p* < 0.001, respectively, between the group and control group of 96 h treatment. (**F**) Morphology of C4-2B cells under treatment of 0, 2.5, 5, 10, 20, 40 µM CAPE for 48 h was shown. Magnification of microscope 10 × 10

 Human C4-2B PCa cells were treated with increasing concentrations of CAPE (0, 2.5, 5, 10, 20, and 40 µM) for 24, 48, 72 and 96 h. Hoechst 33,258 proliferation assay revealed that CAPE dose-dependently suppressed the proliferation of C4-2B cells (Fig. 1A and D). The IC_50_ of CAPE treatment for 24, 48, 72 and 96 h were 57.46 µM, 24.05 µM, 15.08 µM and 12.39 µM, respectively. MTT assay demonstrated that CAPE dose-dependently reduced the cell survival of C4-2B cells. The IC_50_ of CAPE treatment for 48 and 96 h were 15.75 µM and 11.58 µM, respectively (Fig. 1E).

### Oxygen consumption rate (OCR) in C4-2B cells increased after CAPE treatment

**Fig. 2:**
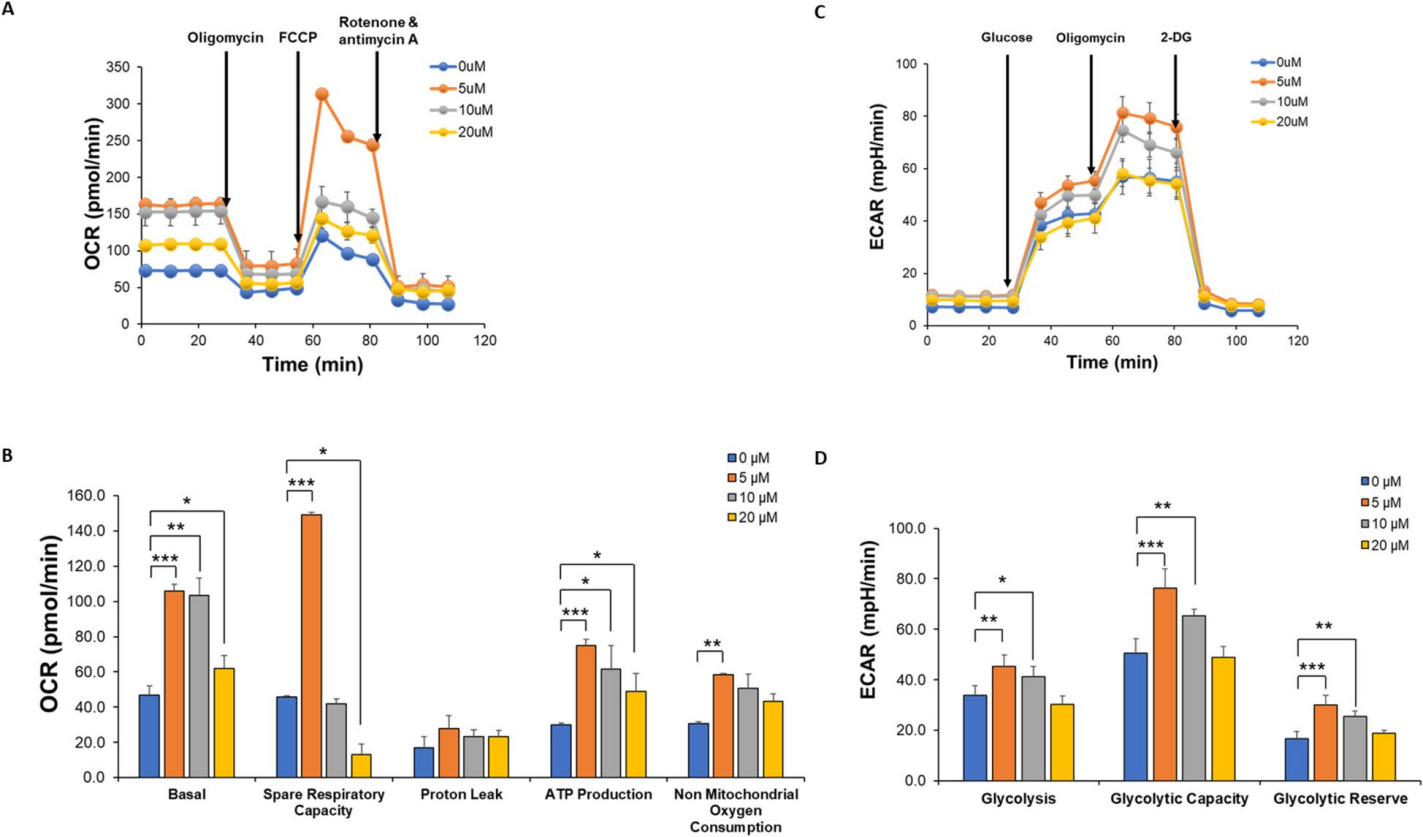
The effects of CAPE treatment on mitochondrial function and glucose metabolism in C4-2B cells. The effects of CAPE on mitochondrial function and glucose metabolism of C4-2B cells were measured by Seahorse Bioscience XFe24 Extracellular Flux Analyzer. C4-2B cells were pretreated with increasing concentration of CAPE (0, 5, 10, 20 µM) for 48 h. Oxygen consumption rate (OCR) (**A**) as well as basal respiration, spare respiratory capacity, proton leak, ATP production and non-mitochondrial oxygen consumption (**B**) of C4-2B cells was shown. Extracellular acidification rate (ECAR) (**C**) and glycolysis, glycolytic capacity as well as glycolytic reserve (**D**) of C4-2B cells were also examined by Seahorse Bioscience XFe24 Extracellular Flux Analyzer. Asterisks *, **, *** represented statistically significant different *p* < 0.05, *p* < 0.01, *p* < 0.001, respectively, between the two groups being compared

 To investigate the effects of CAPE on cell metabolism, we used the Seahorse Bioscience XFe24 extracellular flux analyzer to examine the effect of CAPE on glucose metabolism and mitochondrial function in C4-2B cells. Seahorse Bioscience XFe24 measured the oxygen concentration in the cell supernatant over time and converted the measurement into oxygen consumption rate (OCR) to evaluate mitochondrial respiration in cells. C4-2B cells were pretreated with CAPE (0, 5, 10, and 20 µM) for 48 h. The mitochondrial bioenergetic function was determined by measuring OCR after adding oligomycin, Carbonyl cyanide-4 (trifluoromethoxy) phenylhydrazone (FCCP) and rotenone/antimycin A, sequentially. CAPE significantly increased OCR, especially at the dose of 5 µM (Fig. 2A). Basal respiration, spare respiratory capacity, proton leak, ATP production and non-mitochondrial oxygen consumption of C4-2B cells were also measured (Fig. 2B). CAPE treatment enhanced basal respiration and ATP production of mitochondria in C4-2B cells at all doses. Spare respiratory capacity and non-mitochondrial oxygen consumption were stimulated by 5 µM CAPE, but spare respiratory capacity was repressed by 20 µM CAPE (Fig. 2B).

### Extracellular acidification rate (ECAR) elevated after CAPE treatment

Seahorse analyzer was then applied to evaluate the extracellular acidification rate (ECAR) for glycolysis in C4-2B. The glycolysis stress was measured with the additions of glucose, oligomycin, and 2-deoxyglucose (2-DG). Pretreatment of CAPE at 5 and 10 µM significantly increased the ECAR in C4-2B cells (Fig. 2C). C4-2B cells being pre-treated with 5 or 10 µM of CAPE showed elevated levels of glycolysis, glycolytic capacity, and glycolytic reserve (Fig. 2D).

### Expression of metabolic genes and proteins was altered after CAPE treatment

**Fig. 3:**
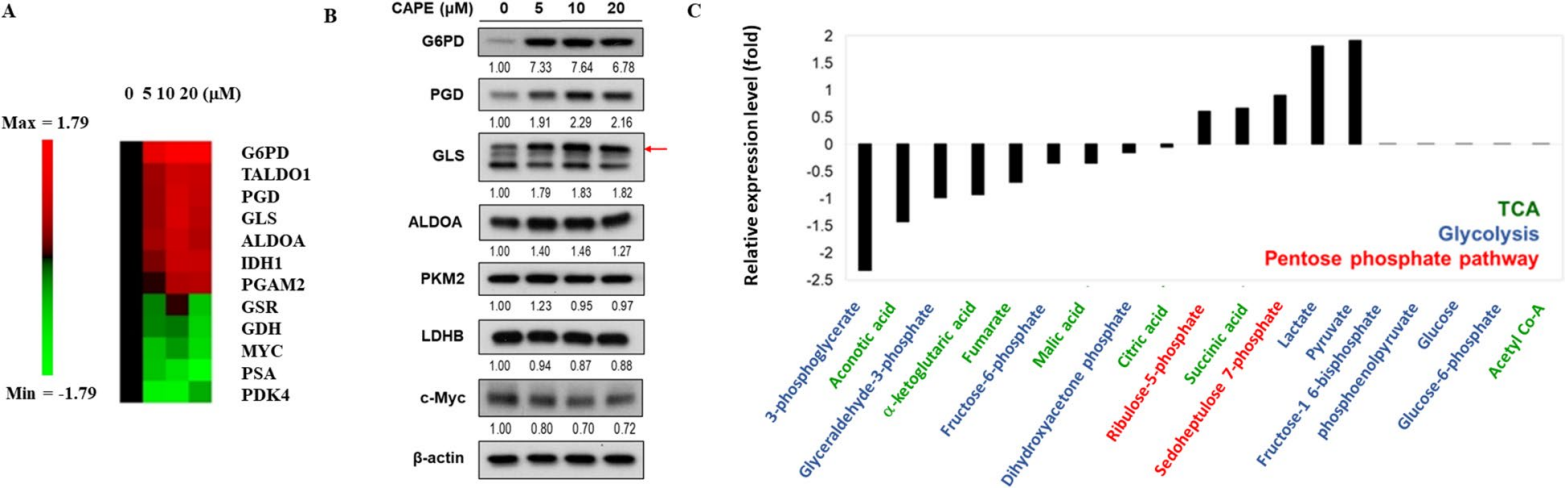
Effects of CAPE treatment on glucose metabolism-related genes and proteins as well as metabolites in C4-2B cells. (**A**) C4-2B cells were treated with increasing concentration of CAPE (0, 5, 10, 20 µM) for 48 h. Total mRNA was extracted and analyzed for the expression of genes involved in glucose metabolism by qRT-PCR. Heatmap indicated the fold change (in log_2_ value) of genes in CAPE-treated cells as compared to control cells. (**B**) Protein expression of G6PD, PGD, GLS, ALDOA, PKM2, LDHB and c-Myc in C4-2B cells being treated with CAPE (0, 5, 10, 20 µM) for 48 h were examined by western blotting. The β-actin was used as loading control. The blots were cropped from different gels but were aligned to be exposed on the same file exposure. (**C**) C4-2B cells were treated with 0 or 20 µM CAPE for 48 h. Cell pellet was collected for analysis of intracellular metabolites by UPLC-MS-Multiple Reaction Monitoring (MRM) in protein chemistry core of NHRI. Green, blue, and red color were used for metabolites involved in TCA cycle, glycolysis, and pentose phosphate pathway, respectively

To determine how CAPE regulates the metabolism, we examined the effect of CAPE treatment on the expression of metabolic genes and proteins in the C4-2B cells after treatment with different concentrations of CAPE (0, 5, 10, and 20 µM) for 48 h (Fig. 3A and B). CAPE up-regulated the gene expression of *G6PD*, *TALDO1*, *PGD*, *GLS*, *ALDOA*, *IDH1* and *PGAM2*, but down-regulated the gene expression of *GSR*, *GDH*, *MYC*, *PDK4* and *PSA* (Fig. 3A). *G6PD* encodes glucose-6-phosphate dehydrogenase, which is responsible to generate NADPH. TALDO1 encodes transaldolase 1, an important enzyme in nonoxidative pentose phosphate pathway to provide ribose-5-phosphate for nucleic acid synthesis and NADPH for lipid biosynthesis. *PGD* encodes for 6-phosphogluconate dehydrogenase, an enzyme in pentose phosphate pathway. *GLS* encodes mitochondrial glutaminase, an essential enzyme generating energy for metabolism. *ALDOA* encodes a class I fructose-bisphosphate aldolase, which catalyzes the reversible conversion of fructose-1,6-bisphosphate to glyceraldehyde 3-phosphate and dihydroxyacetone phosphate. *IDH1* encodes isocitrate dehydrogenases. *PGAM2* encodes for phosphoglycerate mutase which catalyzes the reversible reaction of 3-phosphoglycerate to 2-phosphoglycerate in the glycolytic pathway. *GSR* encodes for glutathione-disulfide reductase. *GDH* encodes glutamate dehydrogenase, which is a mitochondrial matrix enzyme catalyzing the oxidative deamination of glutamate to α-ketoglutarate. MYC encodes the oncoprotein c-Myc. The c-Myc regulates cell cycle, cell survival, stemness as well as some metabolic enzymes, such as LDHA and GLS [42]. *PDK4* gene encodes pyruvate dehydrogenase kinase 4, which locates in the matrix of the mitochondria and can suppress the pyruvate dehydrogenase. *PSA* encodes the prostate specific antigen (PSA) and is a target gene of androgen receptor (AR).

Western blot assay revealed that CAPE treatment at 5–20 µM increased the protein expression of glucose-6-phosphate dehydrogenase (G6PD), phosphogluconate dehydrogenase (PGD), glutaminase (GLS) and Aldolase A (ALDOA), but decreased the protein expression of c-Myc and lactate dehydrogenase B (LDHB) (Fig. 3B).

### Levels of metabolites involved in glucose metabolism were changed in C4-2B cells after CAPE treatment

As we observed that CAPE treatment affected several gene and proteins involved in glycolysis, tricarboxylic acid cycle (TCA cycle) and pentose phosphate pathway (PPP), we used UPLC-MS-Multiple Reaction Monitoring (MRM) to detect the changes of metabolites in C4-2B cells under treatment of control vehicle or 20 µM CAPE. CAPE treatment increased the levels of ribulose-5-phosphate, succinic acid, sedoheptulose-7-phosphate, lactate, and pyruvate as compared to control vehicle in C4-2B cells (Fig. 3C). On the other hand, CAPE treated decreased the levels of 3-phosphoglycerate, aconotic acid, glyceraldehyde-3-phosphate, α-ketoglutaric acid, fumarate, malic acid, dihydroxyacetone phosphate and citric acid (Fig. 3C). To determine if elevation of glucose may affect the proliferation of PCa cells under CAPE treatment, we cultured C4-2B cells in low (11 mM) glucose vs. high (49.5 mM) glucose (new supplemental Fig. 1) with increasing concentration of CAPE. It seemed that the inhibition of proliferation caused by CAPE was not affected by the glucose level in culture medium.

### Intracellular ROS decreased 48 h after CAPE treatment

**Fig. 4:**
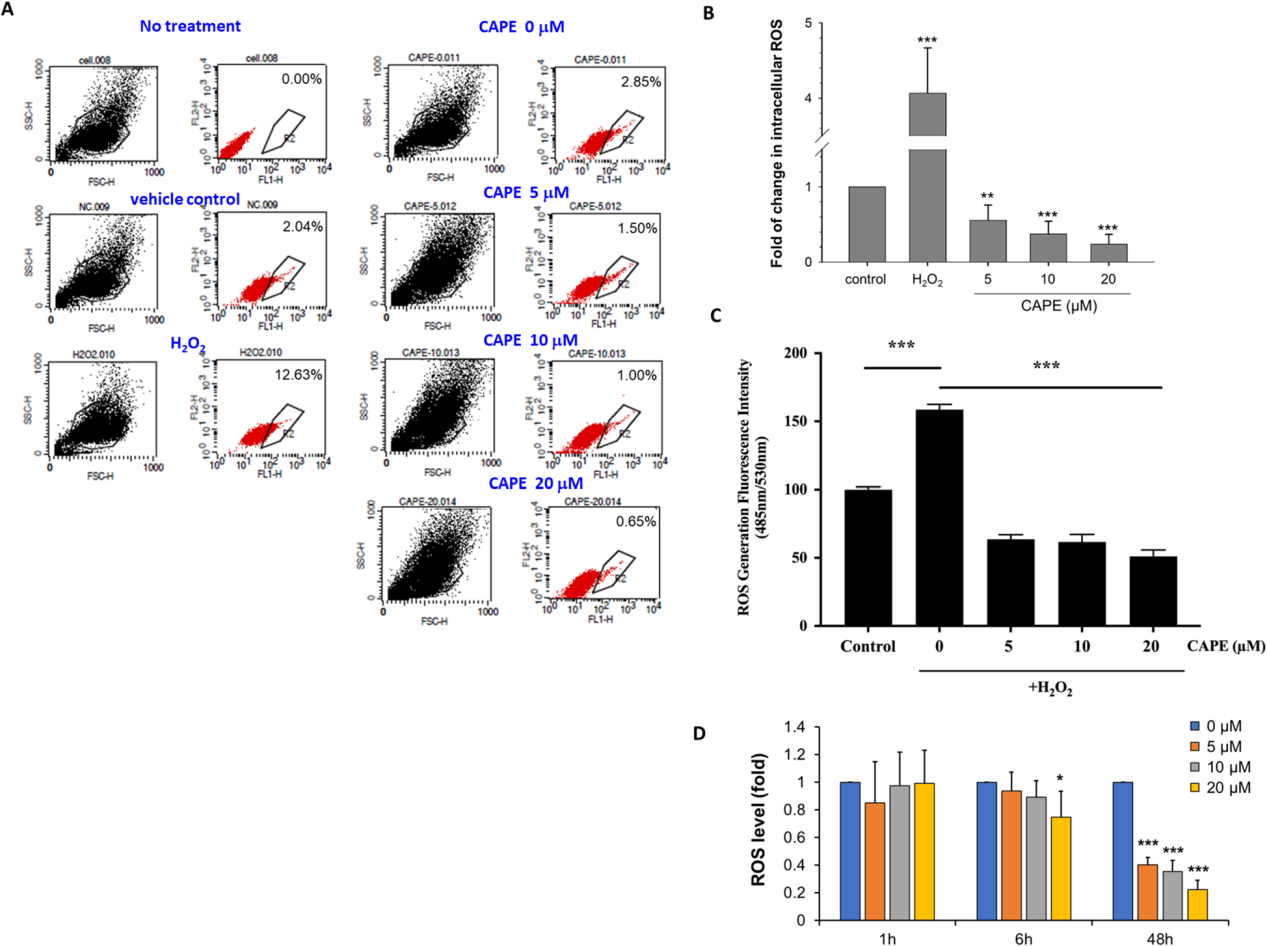
Effects of CAPE treatment on intracellular reactive oxygen species (ROS) of C4-2B cells. **A** C4-2B cells were treated with increasing concentration of CAPE (0, 5, 10, and 20 µM) for 48 h. Cells were then stained with carboxy-H_2_DCFDA for analysis of ROS using flow cytometry. Positive control C4-2B cells were being pre-treated with H_2_O_2_ for 10 min before the analysis. **B** Quantification of intracellular ROS in C4-2B cells being treated with control vehicle, H_2_O_2_, or CAPE (5, 10, and 20 µM) was shown. **C** Intracellular ROS level in C4-2B cells being treated with 1 h, 6 h, 48 h of increasing concentration of CAPE (0, 5, 10, and 20 µM) was determined by ELISA. Asterisks *, **, *** represented statistically significant different *p* < 0.05, *p* < 0.01, *p* < 0.001, respectively, between the specific condition and the control group

Reactive oxygen species (ROS) participate in the modulation of apoptosis, stress responses and cellular proliferation. Excessive ROS can cause damage to cells, while NADPH protects cell against oxidative damage. Glucose-6-phosphate dehydrogenase (G6PD) is one of the important enzymes to produce NADPH [43, 44]. G6PD catalyzes the rate-limiting step in the PPP [44]. To examine if CAPE treatment affects ROS level in C4-2B cells, we used carboxy-H_2_DCFDA to detect the changes of ROS. H_2_O_2_ was used as a positive control to induce intracellular ROS (Fig. 4A and B). Flow cytometry analysis showed the intracellular level of ROS was significantly reduced 48 h after the CAPE treatment (Fig. 4A and B). We next examined the variation of ROS at different times (1 h, 6 h, 48 h) under CAPE treatment (Fig. 4C). After 6 h of CAPE treatment, only the highest concentration of CAPE (20 µM) decreased the ROS. However, all doses of CAPE (5, 10, 20 µM) significantly reduced the ROS level in C4-2B cells 48 h after treatment with CAPE.

### AMPK is activated in C4-2B cells after CAPE treatment

**Fig. 5:**
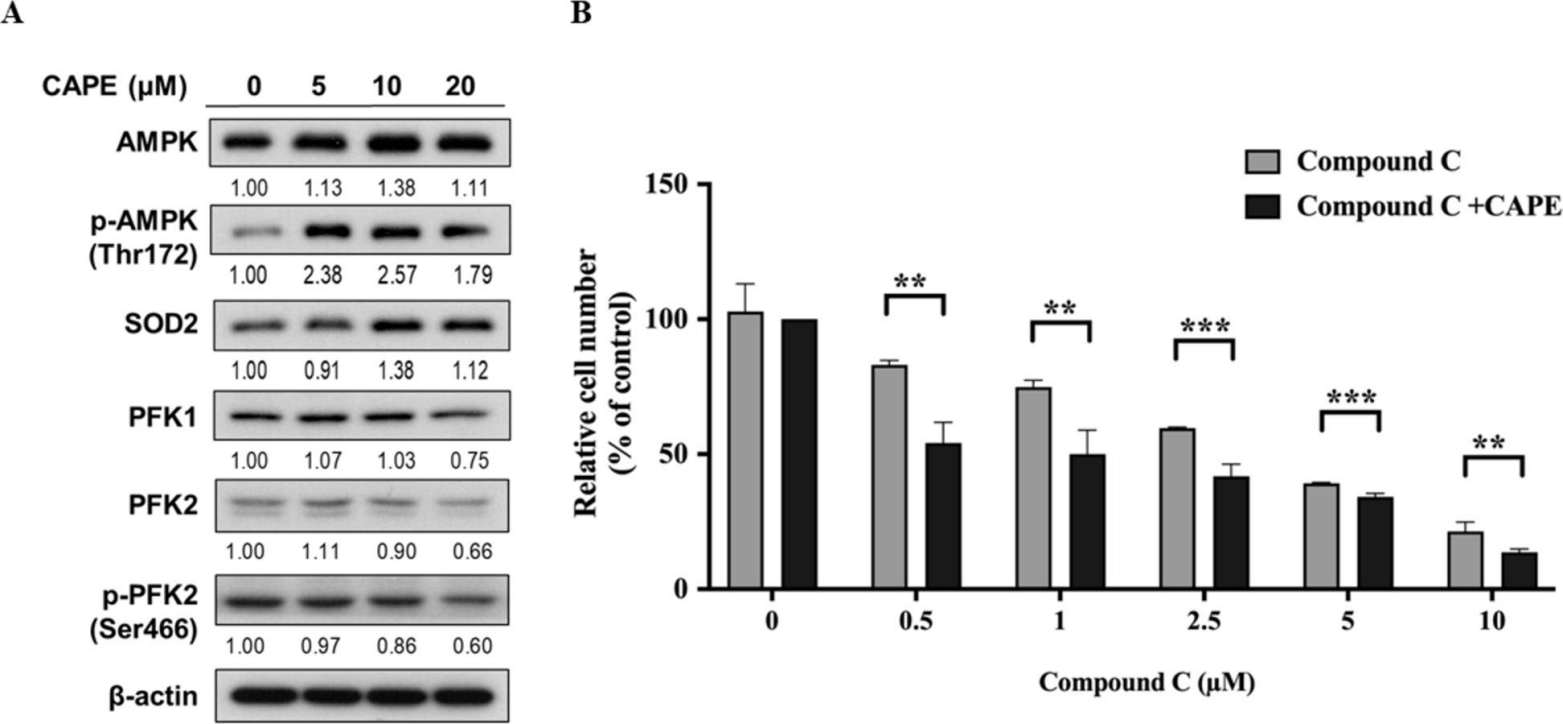
Effects of CAPE treatment on expression of AMPK protein and effects of combined treatment of CAPE with AMPK inhibitor on proliferation of C4-2B cells**.** **A** C4-2B cells were treated with different concentration of CAPE (0, 5, 10, and 20 µM) for 48 h and the protein expression of AMPK, phospho-AMPK Thr172, SOD2, PFK1, PFK2 and phospho-PFK2 Ser466 in C4-2B cells was assayed by western blotting. The β-actin was used as loading control. **B** C4-2B cells were treated with different concentrations (0, 0.5, 1, 2.5, 5, 10 µM) of AMPK inhibitor compound C for 48 h in combination with or without 5 µM CAPE. The cell proliferation was determined by Hoechst 33,258 proliferation assay. We normalized the cell number of C4-2B cells in the combined treatment (CAPE plus compound C) group to the cell number of 5 µM CAPE and set it as 100%. For example, adding 0.5 µM compound C with 5 µM CAPE reduced the cell number to approximately 55% of the cell number under treatment of 5 µM CAPE, which equals to approximately 32% in Fig. 1B. Asterisks *, **, *** represented statistically significant different *p* < 0.05, *p* < 0.01, *p* < 0.001, respectively, between the two groups being compared

Activation of 5’ AMP-activated protein kinase (AMPK) promotes catabolism, increases the ATP synthesis, and maintains balance of energy in cells [45, 46]. AMPK stimulates glycolysis by activating phosphorylation of 6-phosphofructo-2-kinase, fructose-2,6-bisphosphatase 2/3, and glycogen phosphorylase [46]. AMPK inhibits synthesis of fatty acids, cholesterol, and triglycerides via phosphorylating acetyl-CoA carboxylase 1 (ACC1) or sterol regulatory element-binding protein 1c (SREBP1c). Protein levels of both AMPK and phosphorylation on Thr172 of AMPK were increased after CAPE treatment (Fig. 5A). Phosphorylation of Thr172 is required for the activation of AMPK [47]. Treatment with 20 µM CAPE decreased the expressions of PFK1, PFK2 and phospho-PFK2 Ser466 (Fig. 5A). SOD2 is associated with oxidative stress which can eliminate free radicals. Abundance of SOD increased after CAPE treatment, which is possibly a response to ROS stress induced by CAPE. Finally, we examined the effects of co-treatment of AMPK inhibitor compound C and CAPE on proliferation of C4-2B cells. Combination of AMPK inhibitor C and CAPE showed dose-dependent and additive suppressive effects on cell proliferation of C4-2B cells (Fig. 5B).

## Discussion

Glucose is the main energy source for both normal and cancer cells. Catabolism of glucose includes glycolysis, tricarboxylic acid cycle (TCA cycle) and the pentose phosphate pathway (PPP). The catabolism of glucose provides nicotinamide adenine dinucleotide phosphate (NADPH), nucleotides and ATP for synthesis of amino acids and lipid in cells [48]. PPP is important to generate NADPH, which is essential for DNA synthesis and reduction of reactive oxygen species. Up-regulation of glycolysis pathway has been reported to contribute to drug- or therapy- resistance in different types of cancers [26–32]. Treatment with chemotherapy drugs or tyrosine kinase inhibitors have been discovered to induce glycolysis [29, 33, 49–51]. In this study, we demonstrated that CAPE treatment at 2.5–40 µM suppressed the proliferation and survival of PCa cells (Fig. 1). Glycolysis, ECAR, ATP production, and OCR were elevated in C4-2B cells under CAPE treatment (Fig. 2). Interesting, the abundance of most of the metabolites involved in glycolysis was decreased under CAPE treatment, including 3-phosphoglycerate, glyceraldehyde-3-phosphate, fructose-6-phosphate, and dihydroxyacetone phosphate (Fig. 3). However, the levels of pyruvate and lactate increased in C4-2B cells after CAPE treatment (Fig. 3). Increased production of pyruvate and lactate has been shown to promote the development of drug resistance in cancer cells [52–56].

In addition, C4-2B cells upregulated several enzymes involved in glycolysis, TCA cycle and PPP, including G6PD, PGD, ALDOA, TALDO1, GLS and IDH1 (Fig. 3). Protein level of G6PD was increased for 7 folds. G6PD is the first and the rate-limiting enzyme of PPP. G6PD catalyzes glucose-6-phosphate into 6-phosphogluconolactone with the production of NADPH [48]. Overexpression of G6PD is observed in several types of cancer, such as colorectal cancer [57], gastric cancer [58], lung cancer [59], bladder cancer [60], glioma [61], etc. Elevation of G6PD is associated with poor prognosis metastasis, and tumor growth in cancer patients [57, 59–63]. Upregulation of G6PD has been reported to increase therapy- and drug-resistance as well as AKT signaling in cancer cells [57, 60, 64]. C4-2B cells under CAPE treatment also increased the levels of ribulose-5-phosphate and sedoheptulose-7-phosphate (Fig. 3). Both ribulose-5-phosphate and sedoheptulose-7-phosphate are intermediate products in pentose phosphate pathway. Ribulose-5-phosphate can be generated from glycolytic intermediates glucose-6-phosphate. Our observation suggested that the PCa cells upregulated OCR, glycolysis, G6PD, PPP, pyruvate, and lactate in response to CAPE treatment. However, the elevation of glycolysis and ATP production failed to counteract the suppressive effects of CAPE on cellular proliferation and survival.

The ability of CAPE to suppress PI3K-Akt signaling and c-Myc activity in PCa cells may explain its considerable suppressive effects on cellular proliferation and tumor growth of PCa cells [18, 19, 22]. Phosphatase and tensin homolog (PTEN) is a negative regulator for PI3K-Akt signaling. Deletion or mutation of PTEN was detected in 40–70% of PCa patients, resulting in upregulation of PI3K-Akt signaling [65–68]. Enhancement of PI3K-Akt activity is associated with poor clinical outcome in PCa [65, 69–72]. PI3K/Akt pathway regulates glucose uptake and utilization [73]. Activation of PI3K/Akt increases expression of cell surface glucose transporters as well as activates the hexokinase (HK)[74]. HK phosphorylates glucose to retain glucose in cells and activates phosphofructokinase-1 (PFK1)[74]. PFK1 catalyzes the committed step of glycolysis [74]. Activated c-Myc induces the expression of several glycolytic enzymes, including hexokinase 2 (HK2), PFK1, phosphoglycerate kinase-1 (PGK1), lactate dehydrogenase A (LDHA), and pyruvate kinase M2 (PKM2) [75]. CAPE, which significantly suppresses the PI3K-Akt signaling and c-Myc, can therefore effectively decline the proliferation, survival, and tumor growth of PCa cells.

ROS participate in the modulation of apoptosis, stress responses and cellular proliferation. Excessive ROS can induce apoptosis and other damages in both normal and cancer cells. G6PD is one of the important enzymes to produce NADPH, and NADPH is the essential player to protect cell from oxidative damage [43, 44]. We observed that ROS level in C4-2B was reduced 6 h after the treatment of 20 µM CAPE or 48 h after the treatment of 5–20 µM CAPE (Fig. 4). Both the production of NAPDH induced by elevated G6PD (Fig. 3) and the increase of SOD (Fig. 5) can repress ROS in PCa cells.

It is an interesting question whether elevation of glycolysis occurs before or after the suppression of cell proliferation in PCa cells under CAPE treatment. In the current study, we observed that CAPE induced glycolysis 48 h after the treatment according to the evidence of Seahorse analysis platform for mitochondrial glycolysis function (Fig. 2) as well as the changes of genes, proteins, and metabolites assayed by qRT-PCR, western blot, and UPLC-MS-Multiple Reaction Monitoring (MRM) (Fig. 3). The induction of ROS by 10 µM CAPE did not happen within 6 h (Fig. 4). However, we previously reported that treatment of 10 µM CAPE suppressed the phosphorylation of AKT and cell cycle regulatory proteins within 30 min [18]. Additionally, the concentration of glucose in culture medium did not affect the CAPE-induced suppression of cell proliferation (Supplemental Fig. 1). These observations suggested that the CAPE-induced suppression of cell cycle and signaling proteins essential for cell proliferation possibly occured earlier than the induction of ROS and elevation of glycolysis. We noticed that the CAPE-induced proliferation inhibition was dose-dependent (Fig. 1), while the induction of glycolysis was not dose-dependent (Fig. 2). According to our previous studies, treatment with CAPE equal or greater than 20 µM not only repressed proteins regulating cell cycle but also inhibited signaling proteins involved in PI3K/AKT pathways and c-Myc signaling [19]. As c-Myc and AKT signaling also regulagte glucose metabolism, the result of Seahorse analysis platform may reflect the combination effects of induction of glycolysis by 5–20 µM CAPE via G6PD as well as the inhibition of AKT and c-Myc signaling by 20 µM CAPE. As a result, the elevation of glycolysis will not bedose-dependent. On the other hand, the inhibition of AKT and c-Myc signaling is more significant on cell proliferation, therefore a dose-dependent inhibition on cell proliferation under CAPE will be seen.

**Fig. 6:**
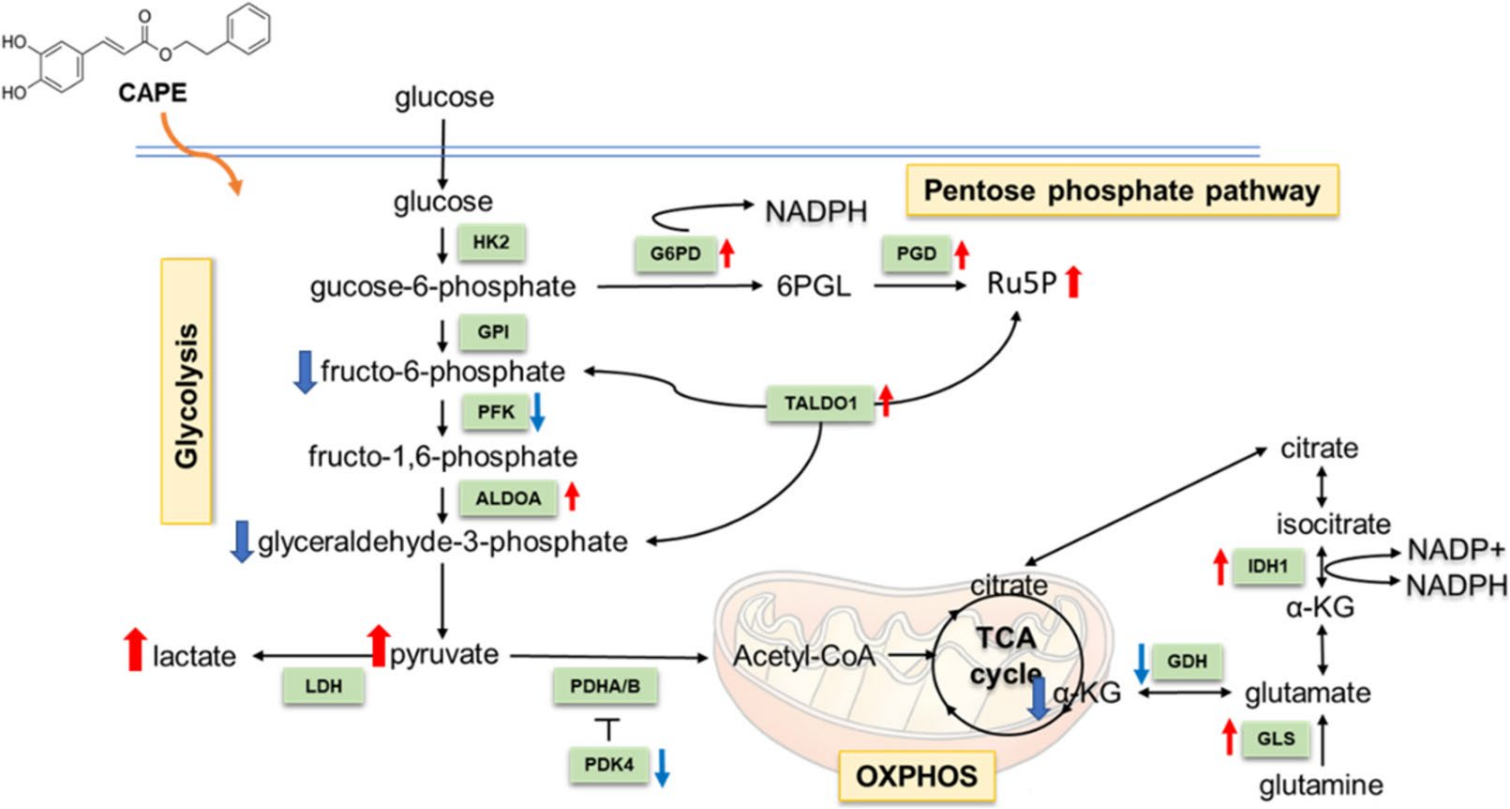
Effects of CAPE treatment on metabolic pathways in C4-2B cells. Effects of CAPE treatment on metabolites and metabolic enzymes observed in current study were shown. Arrows indicated up-regulation or down-regulation on metabolites by CAPE. ALDO, aldolase; ENO, enolase; G6PD, glucose-6-phosphate dehydrogenase; GAPDH, glyceraldehyde 3-phosphate dehydrogenase; GLUTs, glucose transporters; HK, hexokinase; LDH, lactate dehydrogenase; PFK, phosphofructokinase; PGI, phosphoglucose isomerase; PGK, phosphoglycerate kinase; PGM, phosphoglycerate mutase; PK, pyruvate kinase; TCA, tricarboxylic acid cycle; TPI, triose phosphate isomerase; Ru5P, ribulose 5-phosphate

Activation of AMPK promotes catabolism, increases ATP synthesis, and maintains energy balance in cells [45, 46]. AMPK stimulates glycolysis by activating phosphorylation of 6-phosphofructo-2-kinase, fructose-2,6-bisphosphatase 2/3, and glycogen phosphorylase [46]. Total AMPK and phosphorylation of Thr172 on AMPK were increased 48 h after CAPE treatment (Fig. 5), which contributed to the upregulation of glycolysis in response to CAPE-induced growth inhibition and stress. In our current and previous studies, we demonstrated that CAPE treatment can suppress the proliferation of PCa cells. In this study, we showed that CAPE treatment induced activation of AMPK, possibly due to the stress-induced by CAPE (Fig. 5). We therefore hypothesized that upregulation of AMPK partially hindered the suppressive effect of CAPE on proliferation. To prove this hypothesis, we compared the suppressive effect of compound C alone with the suppressive effect of combined treatment of CAPE and compound C. We believed that treatment with compound C with CAPE will show additive suppressive effect compared to compound C alone or CAPE alone. This is because that compound C can repress AMPK, and simultaneous inhibition of AMPK in CAPE-treated PCa cells can repress the CAPE-induced AMPK and thus assist the growth inhibition. The fact that co-treatment of AMPK inhibitor with CAPE demonstrated additive suppressive effect on proliferation of C4-2B cells (Fig. 5) supported the hypothesis that C4-2B cells upregulated glycolysis in response to the stress and growth inhibition caused by CAPE. Combined treatment showed greated inhibition on proliferation of C4-2B cells as compared to copound C treatment alone or CAPE treatment alone (Fig. 5). Simultaneous treatment of CAPE and glycolysis inhibitors may cause greater inhibition on survival and proliferation of CRPC tumors in PCa patients. We summarized the proposed mechanism in our current study in Fig. 6.

## Conclusions

In conclusion, we demonstrated that PCa cells attempted to overcome the CAPE-induced stress and reactive oxygen species (ROS) by upregulation of glycolysis and G6PD but failed to impede the growth inhibition caused by CAPE. Co-treatment of AMPK inhibitor with CAPE exhibited additive growth inhibition on PCa cells. Concurrent treatment of CAPE and inhibitors targeting glycolysis may be promising therapy for advanced PCa.

## Supplementary Information

Additional file 1.

Additional file 2.

Additional file 3. The effect of different glucose concentration in culture medium on cell proliferation of C4-2B cells under CAPE treatment. Human C4-2B PCa cells were treated with increasing concentrations of CAPE (0, 5, 10, 20 μM) for 48 h in the presence of 11 mM or 49.5 mM glucose in culture medium. The cell proliferation was examined by Hoechst 33258 proliferation assay. Relative cell number of each condition was normalized to the cell number of cells under treatment of 11 mM gluocse. Asterisk*** represented statistically significant difference of *p* < 0.001 as examined by One-Way ANOVA.

## Data Availability

All data generated or analyzed during this study are included in this published article and its supplementary information files.

## Abbreviations

ALDOA: Aldolase A
AMPK: 5’ AMP-activated protein kinase
AR: androgen receptor
CAPE: caffeic acid phenethyl ester
CRPC: castration-resistant prostate cancer
ECAR: extra cellular acidification rate
EMT: epithelial-mesenchymal transition
FCCP: Carbonyl cyanide-4 (trifluoromethoxy) phenylhydrazone
G6PD: glucose-6-phosphate dehydrogenase
GLS: glutaminase
HK2: hexokinase 2
LDH: lactate dehydrogenase
NAPDH: nicotinamide adenine dinucleotide phosphate
OCR: oxygen consumption rate
PCa: prostate cancer
PGD: phosphogluconate dehydrogenase
PGK1: phosphoglycerate kinase-1
PFK1: phosphofructokinase-1
PKM2: pyruvate kinase M2
PPP: pentose phosphate pathway
PSA: prostate specific antigen
PTEN: phosphatase and tensin homolog
ROS: reactive oxygen species
TCA cycle: tricarboxylic acid cycle
TKI: tyrosine kinase inhibitor
