# Supplementary material for: Prostate cancer cells elevate glycolysis and G6PD in response to caffeic acid phenethyl ester-induced growth inhibition
Source: BMC Cancer. 2025 Jan 16;25:95. doi: 10.1186/s12885-025-13477-6 (PMC11737093; doi:10.1186/s12885-025-13477-6)
Supplement: Supplementary file 1 — Additional file 1. [file 12885_2025_13477_MOESM1_ESM.docx]

Supplemental Materials and Methods

Seahorse Bioenergetics Analysis

Seahorse Extracellular Flux Analyzer (Seahorse Bioscience) was used to monitored cellular oxygen consumption rates (OCR) and extracellular acidification rates (ECAR) in real-time by Mito Stress Test and Glycolysis Stress Test, respectively. LNCaP C4-2B cells were planted at a density of 1×10^6^ cells in every 10-cm dishes. After one day, cells were pretreated in different concentrations (0, 5, 10, 20 µM) for 48 h. Cells were harvested and re-suspended to re-seed at a density of 3×10^4^ cells per well in a 24-well XF cell culture microplate (Seahorse Bioscience) in 250 μL of RPMI-1640 culture medium, incubated at 37°C with 5% CO_2_ overnight. On the day of assay, microplate was removed from the incubator and centrifuged at 500 rpm for 30 seconds. Removing all the culture medium and replaced with 675 μL assay medium which contains 2% FBS but without sodium bicarbonate (pH7.4) for the Mito Stress Test. In addition, in Glycolysis Stress Test the assay medium contained 2% FBS but without sodium bicarbonate and glucose (pH7.4). Placed the 24-well XF cell culture microplate at 37°C with a non-CO_2_ incubator for 1 hour prior to the assay. OCR values were first measured at baseline and being measured successively after drug injection. The injection volume of the drug injection tank is 75, 85, 95, 100 μL in the order. We evaluated the basic energy metabolism parameters of mitochondrial function in the Mito Stress Test and sequentially added the reagents of oligomycin (1 μM), FCCP (0.5 μM), and rotenone/antimycin A (0.5 μM). ECAR values and the glycolytic parameters were measured at baseline and being measured after sequential injections of glucose (10 mM), oligomycin (1 μM), and 2-deoxyglucose (2-DG) (50 mM). OCR and ECAR values were monitored during each measurement cycle, which consisted of mix-wait-measure times of 3min-2min-3min by a Seahorse XF24 Analyzer.

Measurement of intracellular oxidative stress

We used the ROS indicator 6-carboxy-2’,7’-dichlorodihydrofluorescein diacetate (carboxy-H_2_DCFDA, C400; Invitrogen) to assess the intracellular ROS generation after CAPE treatment. Flow cytometry was used for analysis. Carboxy-H_2_DCFDA, oxidation insensitive, a general oxidative stress indicator, which has a cell-permeable nonfluorescent probe can pass the cell membrane and de-esterified by intracellular esterase to the nonfluorescent polar derivative H_2_DCF. The intracellular ROS oxidize the H_2_DCF to a highly fluorescent compound, DCF. LNCaP C4-2B cells were seeded at a density of 1×10^6^ cells in the 10-cm culture dish and treated with CAPE (0, 5, 10, 20 µM) for 48 h. The cells treated with 0.03% H_2_O_2_ for 10 min was used to be a positive control. After treatment, cells were collected by trypsin and centrifuged for 4 min at 1,000 rpm. Washed and suspension cell in PBS prior to the assay. Immediately prior to use, prepare a fresh stock solution of carboxy-H_2_DCFDA in DMSO. To evaluate the intracellular ROS levels, cells were resuspended in 5 µM carboxy-H_2_DCFDA in serum-free RPMI 1640 medium and incubation in the dark for 30 minutes at 37°C with 5% CO_2_ ^35^. After 30 minutes, removed the carboxy-H_2_DCFDA and the cells were washed and resuspended in PBS. The intracellular ROS was determined by flow cytometry and analyzed with FlowJo software. On the other hand, we also measured intracellular ROS by carboxy-H_2_DCFDA after CAPE treatment for different time (1 h, 6 h, and 48 h) and monitored by using a microplate reader. The fluorescence could be detected by using excitation wavelength at 488 nm and emission wavelengths at 520 nm that were appropriated for green fluorescence.
