# Supplementary material for: Prostate cancer cells elevate glycolysis and G6PD in response to caffeic acid phenethyl ester-induced growth inhibition
Source: BMC Cancer. 2025 Jan 16;25:95. doi: 10.1186/s12885-025-13477-6 (PMC11737093; doi:10.1186/s12885-025-13477-6)
Supplement: Supplementary file 2 — Additional file 2. [file 12885_2025_13477_MOESM2_ESM.pdf]

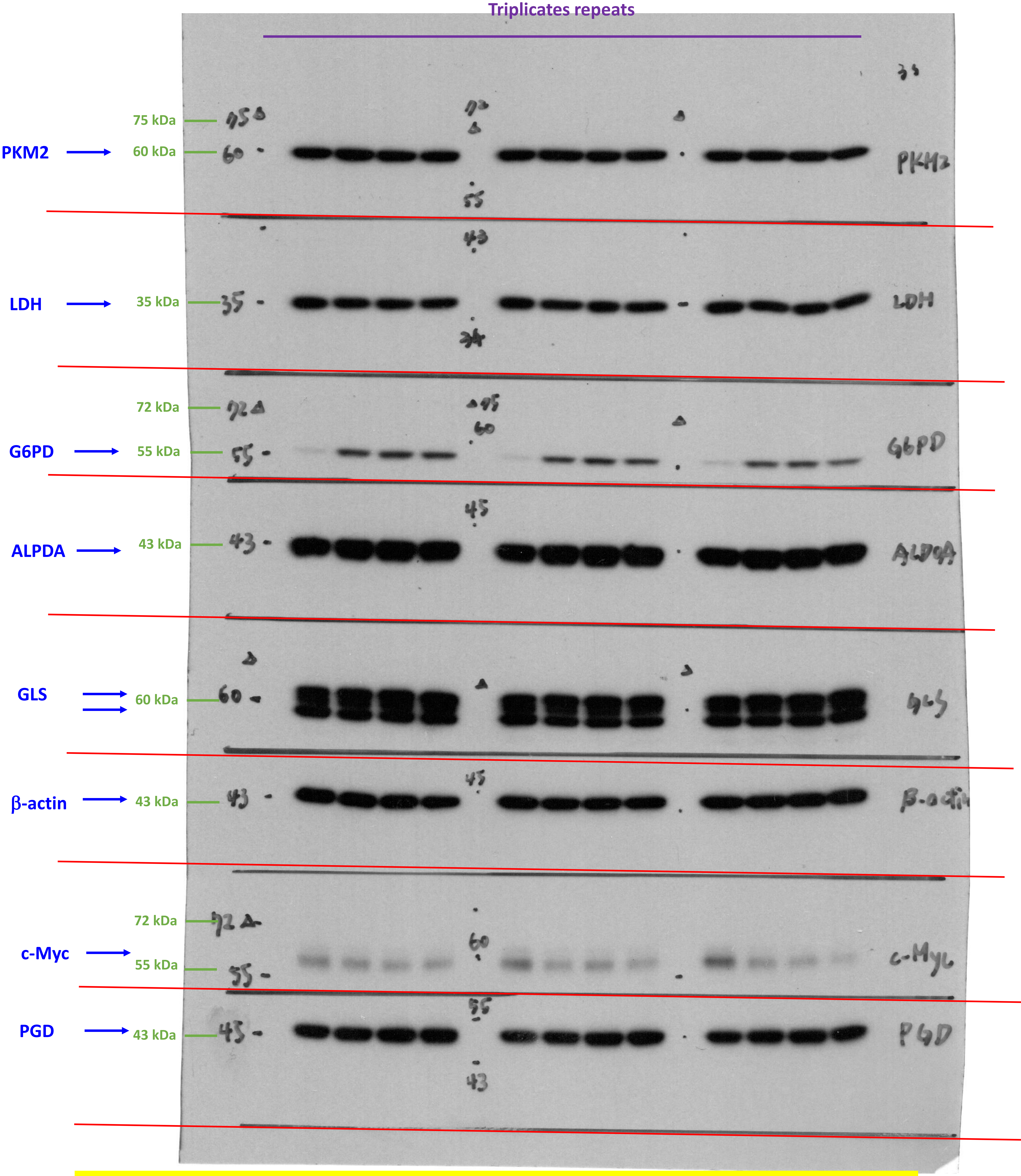

Original film image of western blot assay for PKM2, LDH, G6PD, ALPDA, GLS, c-Myc, PGD. The β-acin was used as loading control.

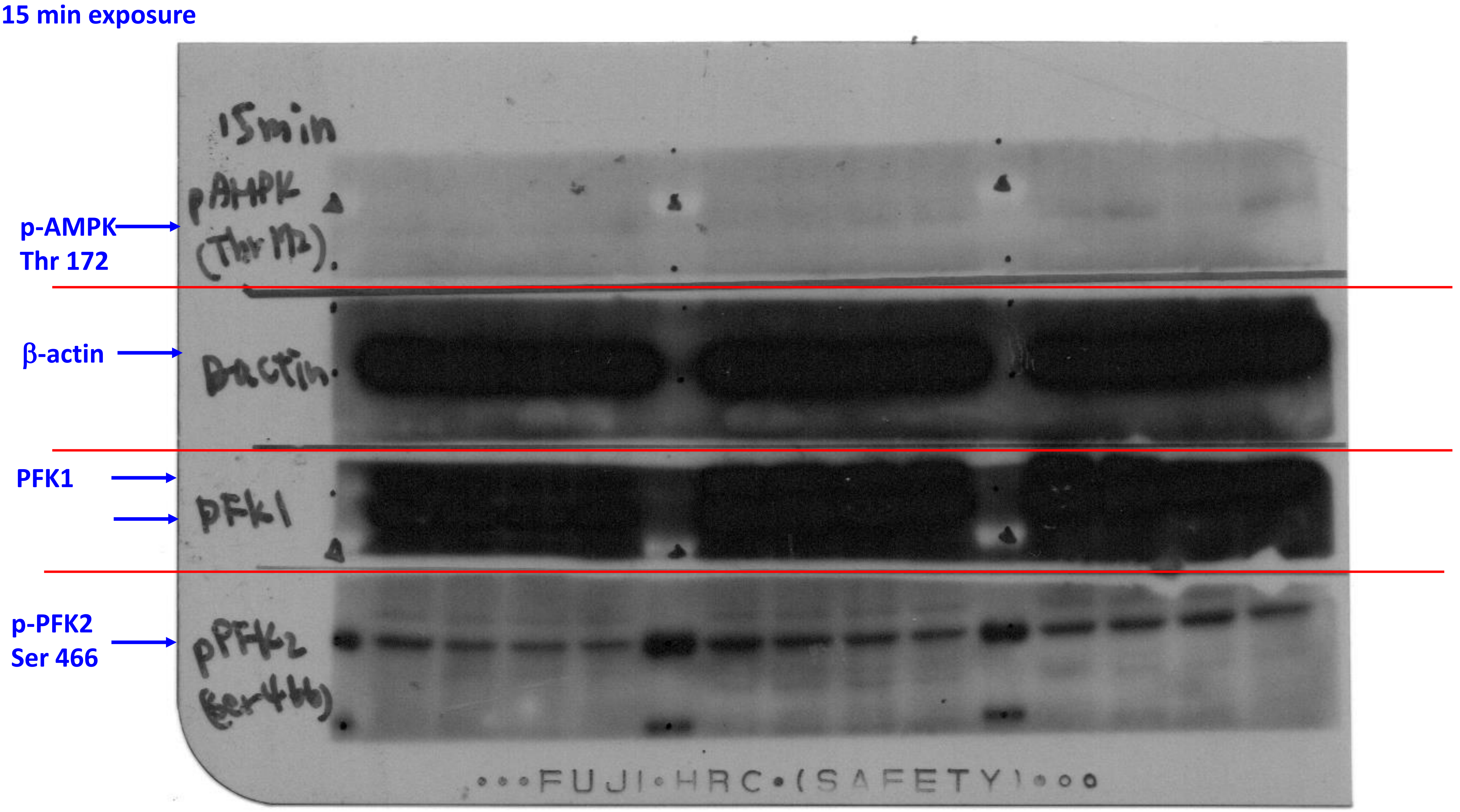

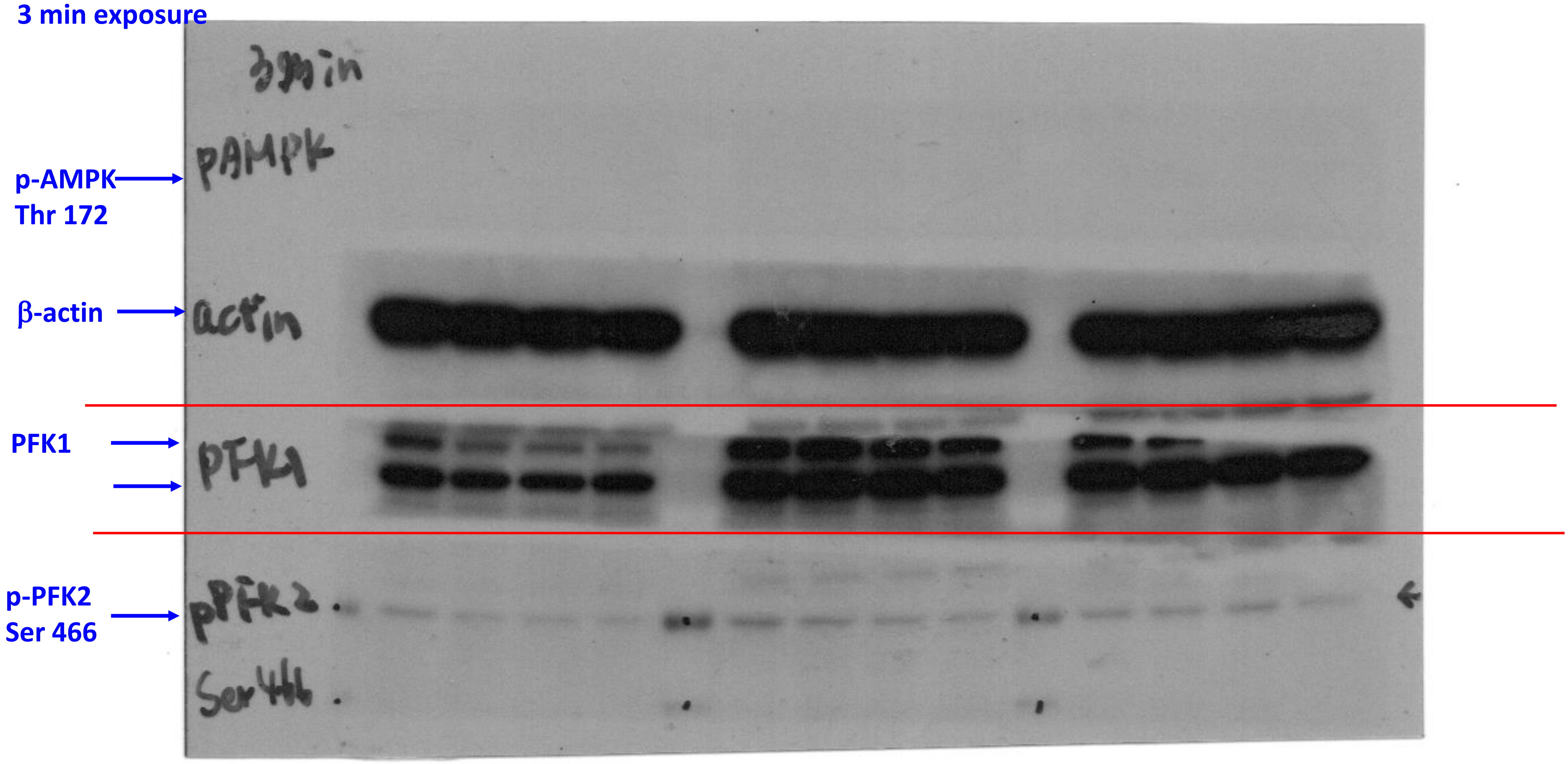

Original film image of western blot assay for p-AMPK Thr 172, PFK1, p-PFK2 Ser 466. The β-acin was used as loading control. The two films were the same blot with different exposure time.

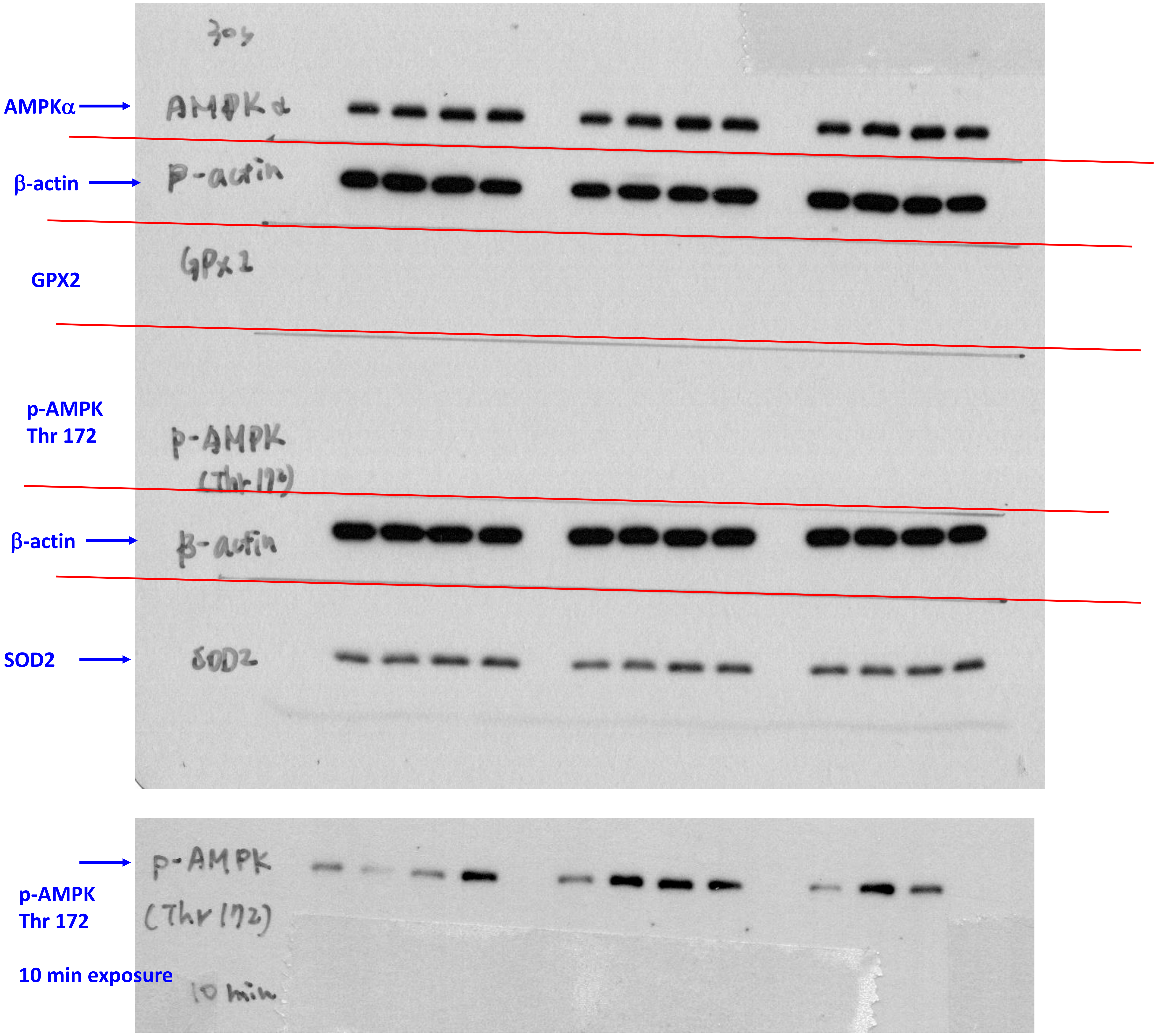

Original film image of western blot assay for GPX2, p-AMPK Thr 172, SOD2, AMPKα. The β-acin was used as loading control.
