# Supplementary material for: Prostate cancer cells elevate glycolysis and G6PD in response to caffeic acid phenethyl ester-induced growth inhibition
Source: BMC Cancer. 2025 Jan 16;25:95. doi: 10.1186/s12885-025-13477-6 (PMC11737093; doi:10.1186/s12885-025-13477-6)

**Supplemental Figure 1. The effect of different glucose concentration in culture medium on cell proliferation of C4-2B cells under CAPE treatment.** Human C4-2B PCa cells were treated with increasing concentrations of CAPE (0, 5, 10, 20 μM) for 48 h in the presence of 11 mM or 49.5 mM glucose in culture medium. The cell proliferation was examined by Hoechst 33258 proliferation assay. Relative cell number of each condition was normalized to the cell number of cells under treatment of 11 mM gluocse. Asterisk *** represented statistically significant difference of *p* < 0.001 as examined by One-Way ANOVA.


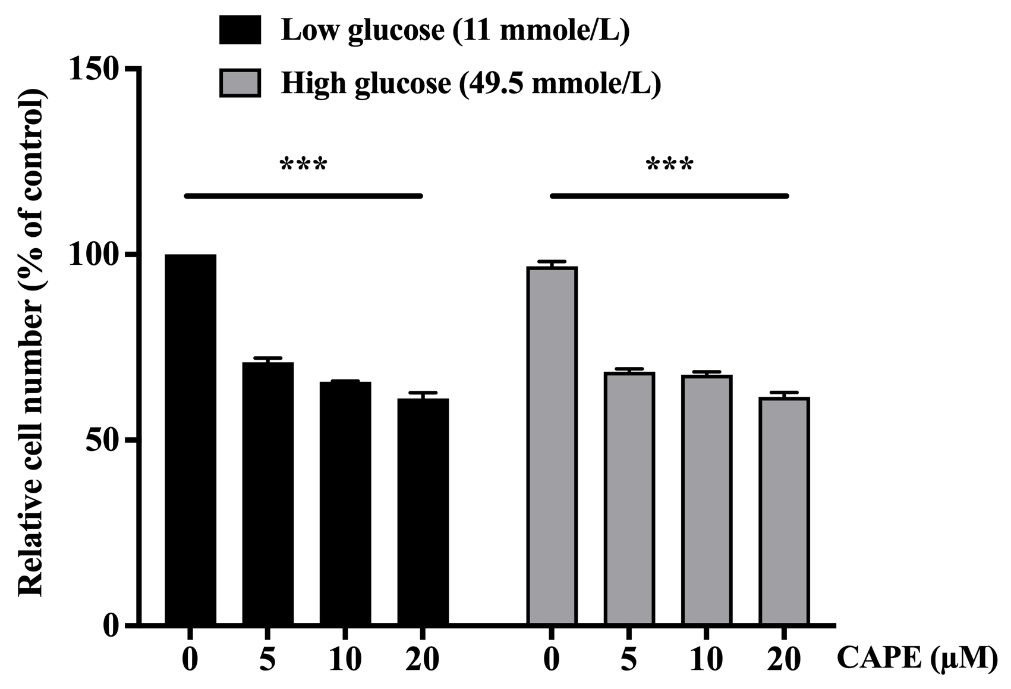

Supplement: Supplementary file 3 — Additional file 3. The effect of different glucose concentration in culture medium on cell proliferation of C4-2B cells under CAPE treatment. Human C4-2B PCa cells were treated with increasing concentrations of CAPE (0, 5, 10, 20 μM) for 48 h in the presence of 11 mM or 49.5 mM glucose in culture medium. The cell proliferation was examined by Hoechst 33258 proliferation assay. Relative cell number of each condition was normalized to the cell number of cells under treatment of 11 mM gluocse. Asterisk*** represented statistically significant difference of p < 0.001 as examined by One-Way ANOVA. [file 12885_2025_13477_MOESM3_ESM.docx]
